# A novel missense single-nucleotide polymorphism (c.149G>A) in the bovine *leptin* gene and its association with growth traits in Madura cattle

**DOI:** 10.14202/vetworld.2025.2072-2077

**Published:** 2025-07-27

**Authors:** Kuswati Kuswati, Irida Novianti, Rizki Prafitri, Wike Andre Septian, Rafika Febriani Putri, Chairdin Dwi Nugraha, Ahmad Furqon

**Affiliations:** 1 Department of Animal Science, Faculty of Animal Science, University of Brawijaya, Veteran Street, Malang City, Indonesia; 2 Research Center for Animal Husbandry, National Research and Innovation Agency, Republic of Indonesia, Bogor, Indonesia; 3 Research Center for Applied Zoology, National Research and Innovation Agency, Republic of Indonesia, Bogor, Indonesia

**Keywords:** c.149G>A, growth traits, *leptin* gene, Madura cattle, missense mutation, molecular marker, single-nucleotide polymorphism

## Abstract

**Background and Aim:**

The *leptin* (*LEP*) gene plays a pivotal role in regulating growth, metabolism, and fat deposition in cattle. Genetic polymorphisms in this gene can influence phenotypic traits and may serve as molecular markers for selection in breeding programs. However, comprehensive characterization of *LEP* gene variants in local Indonesian breeds, such as Madura cattle, remains limited. This study aimed to identify novel single-nucleotide polymorphisms (SNPs) within exon 2 of the bovine *LEP* gene and assess their association with growth traits in Madura cattle.

**Materials and Methods:**

Forty-five Madura cows (aged 2–4 years) were phenotypically evaluated for body weight (BW), wither height (WH), body length (BL), chest girth (CG), hip height (HH), head length (HL), and head width (HW). Genomic DNA was extracted and subjected to polymerase chain reaction amplification followed by Sanger sequencing. Detected SNPs were analyzed for genotype and allele frequencies, Hardy–Weinberg equilibrium (HWE), and their associations with growth traits using a general linear model.

**Results:**

Three SNPs were identified in exon 2 of the *LEP* gene: c.126T>C (synonymous), c.148T>C (missense), and a novel missense SNP c.149G>A, resulting in a cysteine to tyrosine substitution at amino acid position 50. The c.149G>A SNP showed polymorphism with three genotypes (GG, GA, AA), and the heterozygous GA genotype had the highest frequency (64.4%). This SNP deviated from HWE (p < 0.05), indicating potential selection pressure or population structure effects. While no statistically significant associations were found between genotypes and growth traits (p > 0.05), the AA genotype showed the highest mean values across most body measurements.

**Conclusion:**

Although the novel c.149G>A SNP was not significantly associated with growth traits, its polymorphic nature and descriptive trait patterns suggest it may have biological relevance. Larger-scale studies are recommended to validate its utility as a genetic marker for growth and development in Madura cattle.

## INTRODUCTION

Madura cattle generally reach a peak body weight (BW) of 284.75 kg, a body length (BL) of 127.72 cm, a height of 123.86 cm, and a chest girth (CG) of 156.26 cm by the age of 2–3 years [1]. They exhibit a relatively high carcass yield, averaging 52.22% in cows and 52.84% in bulls [1]. Their rapid BW gain results from the interplay of multiple genes and hormones. Cattle growth is a multifactorial process that influences both physical development and metabolic functions. Several key genes involved in regulating growth include growth hormone (GH), GH receptor, myostatin, insulin-like growth factor 1, and *leptin* (*LEP*) [2, 3].

The *LEP* gene plays a crucial role in various physiological functions, including growth, BW regulation, appetite control, energy metabolism, fat storage, immune function, and reproductive capability [4]. *LEP*, a hormone secreted by adipose tissue, enters the bloodstream and helps regulate BW by curbing appetite [5]. Moreover, *LEP* supports energy balance by modulating glucose and lipid metabolism, which in turn promotes optimal growth in cattle [6]. Due to these functions, the *LEP* gene is considered a promising candidate marker for growth and carcass-related traits. Located on chromosome 4, the bovine *LEP* gene encodes a 16 kDa protein consisting of 146 amino acids [7]. Structurally, the gene comprises three exons and two introns that contribute to the formation of the *LEP* protein [8]. Once secreted into circulation, *LEP* binds to carrier proteins and is transported to the brain, where it interacts with receptors in the hypothalamus to trigger satiety signals and regulate food intake [9].

Genetic variations in the *LEP* gene have been linked to traits such as growth performance, carcass characteristics, and milk production in Madura and other Indonesian cattle breeds, underscoring its value as a molecular marker for selection [6]. The use of such markers in breeding programs can significantly accelerate genetic improvement [10]. A prior study identified exon 2 polymorphisms in the *LEP* gene using the restriction fragment length polymorphism (RFLP) method with the AciI restriction enzyme [4].

Although previous studies have identified single-nucleotide polymorphisms (SNPs) in the *LEP* gene and established their associations with growth traits in various cattle breeds, there is a limited understanding of the specific genetic variations in the *LEP* gene of Madura cattle, a local Indonesian breed valued for its adaptability and meat quality. Most prior investigations have employed preliminary genotyping techniques, such as RFLP, which may not detect novel mutations or provide comprehensive information on functional SNPs. Furthermore, the association between newly emerging missense mutations in exon regions of th*e LEP* gene and detailed phenotypic traits in Madura cattle has not been thoroughly explored. This lack of high-resolution genetic profiling restricts the development of breed-specific molecular markers, which are essential for effective selection programs.

This study aimed to identify novel SNP polymorphisms within exon 2 of the *LEP* gene using Sanger sequencing and assess their association with BW and linear body measurements in Madura cattle. By analyzing the genotypic and allelic distributions and evaluating their potential functional effects, this research seeks to provide foundational genetic insights that could support future molecular-assisted breeding strategies to improve growth performance in this indigenous breed.

## MATERIALS AND METHODS

### Ethical approval

All experimental procedures involving animals were conducted in accordance with ethical standards and approved by the Animal Care and Use Committee of Universitas Brawijaya (Approval No. 019-KEP-UB-2021).

### Study period and location

This study was conducted in April 2020 at Village Breeding Center (VBC) in Waru, Pamekasan, Madura for collecting sample. Molecular analysis was performed from May 2023 to August 2023 at the Laboratory of Animal Biotechnology, Faculty of Animal Science, University of Brawijaya, Malang, Indonesia.

### Animal management and phenotypic data collection

A total of 45 Madura cows, aged between 2 and 4 years, were obtained from the Village Breeding Center in Waru, Madura Island, Indonesia. The animals were group-housed under traditional farming conditions and maintained on a uniform feeding and management regime.

Blood samples were taken from the jugular vein of each animal into 5 mL ethylenediaminetetraacetic acid tubes, immediately mixed, and stored at 4°C for later genetic analysis. Morphometric traits measured included BW, WH, BL, CG, hip height (HH), head length (HL), and head width (HW). These measurements were recorded using a measuring stick and tape measure, following standard protocols [11].

### Genomic DNA extraction and polymerase chain reaction (PCR) amplification

Genomic DNA was extracted from 0.2 mL of whole blood using the Genomic DNA Mini Kit (Blood/Cultured Cells; Geneaid Biotech Ltd., China). DNA integrity was verified via 1.5% agarose gel electrophoresis, stained with Diamond Nucleic Acid Dye (Promega, USA), and concentration was determined using a NanoDrop ND-1000 spectrophotometer (Thermo Fisher Scientific, USA). Each DNA sample was diluted to a final concentration of 50 ng/μL for amplification.

Specific primers were designed to amplify a 267-bp region within exon 2 of the bovine *LEP* gene based on GenBank reference sequence U50365.1 [4]. The forward primer sequence was 5′-CAT CTG AAG ACG TGG ATG CG-3′ and the reverse primer was 5′-CCT ACC GTG TGT GAG ATG TC-3′. PCR was performed in a 30 μL reaction mix consisting of 50 ng/μL DNA template, 10 pmol/μL of each primer, and 1× GoTaq Green Master Mix (Promega). The thermal cycling conditions included an initial denaturation at 94°C for 5 min, followed by 35 cycles of denaturation at 94°C for 10 s, annealing at 60°C for 20 s, and extension at 72°C for 30 s, with a final extension at 72°C for 5 min.

PCR products were resolved on 1.5% agarose gels, stained with Diamond Nucleic Acid Dye (Promega), and visualized under blue light using a Glite 965 GW imaging system (Pacific Image Electronics Co., Ltd., Taiwan).

### DNA sequencing and SNP genotyping

Thirty microliters of purified PCR product from each sample were submitted for Sanger sequencing at 1^st^ BASE DNA Sequencing Services (Selangor, Malaysia). The resulting sequences were aligned using MEGA 11 software (https://mega.io/desktop) and compared to the bovine *LEP* gene reference sequence (GenBank accession number U50365.1). SNP sites were identified through alignment, and genotyping was performed for three polymorphisms: c.126T>C, c.148T>C, and the novel c.149G>A.

### Statistical and association analysis

Genotype and allele frequencies were calculated by direct counting. Hardy–Weinberg equilibrium (HWE) for each SNP locus was assessed using Chi-square tests. A randomized complete block design was employed to analyze the effect of genotype on growth traits, with age as the blocking factor to minimize variability.

A general linear model in SPSS version 26.0 (IBM Corp., NY, USA) [11, 12] was used to assess the association between *LEP* gene polymorphisms and phenotypic traits (BW, WH, BL, CG, HH, HL, and HW). Genotype was treated as the fixed effect, and age was included as a covariate to improve statistical accuracy.

## RESULTS

### SNP identification

Three SNP polymorphisms were identified in the *LEP* gene of Madura cattle (c.126T>C, c.148T>C, and a novel missense SNP c.149G>A), as presented in Table 1. SNP 1 with the nucleotide sequence CTGTC (T/C) TACGT located at the 126^th^ nucleotide based on the coding region, with a base change from T to C. This synonymous mutation did not alter the amino acid sequence. SNP 2 with the nucleotide sequence CCATC (C/T) GCAAG located at the 148^th^ nucleotide based on the coding region, with a change in base T to C. This type of mutation is a missense mutation, changing the amino acid from cysteine (Cys) to Arginine (Arg) at the 50^th^ position. SNP 3 with the nucleotide sequence CATCC (G/A) CAAGG, located at the 149^th^ nucleotide based on the coding region, with a change in the base G to A. This mutation was classified as a missense variant, substituting Cys with tyrosine (Tyr) at position 50. The third SNP is a novel finding because it has not yet been assigned a reference number.

**Table 1:** SNP identification.

SNPs	Location	Mutation type	Amino acid changes	RS number
CTGTC (T/C) TACGT	c.126T>C	Synonymous	p. Ser42=	rs520148521
CCATC (C/T) GCAAG	c.148T>C	Missense	p. Cys50Arg	rs29004488
CATCC (G/A) CAAGG	c.149G>A	Missense	p. Cys50Tyr	Novel

SNPs=Single-nucleotide polymorphisms, Cys=Cysteine, Arg=Arginine, Tyr=Tyrosine, RS=Reference SNP, Ser=Serin

### Polymorphism of the *LEP* gene in exon 2

Table 2 shows the polymorphisms of the *LEP* gene in Madura cattle. At the SNP1 locus, two alleles (T and C) were detected, resulting in two genotypes (TT and TC) in the population. The most frequent genotype was TT, whereas the T allele had the highest frequency. A similar pattern was observed at the SNP2 locus, with two alleles (T and C) forming two genotypes TC and CC. The CC genotype had the highest frequency with the C allele. This C allele frequency was found to be more than 0.750 in the cattle population. The polymorphism of SNP3 showed more diverse results with the presence of two main alleles and three genotypes in the Madura cattle population. Three genotypes (GG, GA, and AA) were identified at the SNP3 locus.

**Table 2:** Frequencies of genotypes and alleles of the *LEP* gene (c.126T>C; c.148T>C; c.149G>A) polymorphism in Madura cattle.

SNPs	n	Genotype frequencies			Allele frequencies		Chi-square value
SNP1 (c.126T>C)	45	TT	TC	CC	T	C	1.527
		0.689	0.311	0	0.844	0.156	
SNP2 (c.148T>C)	45	TT	TC	CC	T	C	1.527
		0	0.311	0.689	0.156	0.844	
SNP3 (c.149G>A)	45	GG	GA	AA	G	A	4.387*
		0.244	0.644	0.111	0.567	0.433	

* Significant, χ² (α 0.05;1)=3.84. SNPs=Single-nucleotide polymorphisms, n = Number of samples, LEP=Leptin

Based on the Chi-square analysis, SNP1 and SNP2 showed that the Chi-square value (1.527) was smaller (p > 0.05) than the Chi-square table (3.84). The Madura cattle population was in HWE for SNPs 1 and 2. A different result was observed for SNP3, where the Madura cattle population was not in equilibrium with the HWE. This was indicated by the Chi-square value (4.387) being higher (p < 0.05) than the Chi-square table value (3.84). HWE analysis was incorporated to validate the genetic structure of the studied population and to ensure the reliability of association analyses.

### Association between novelty SNP (c.149G>A) genotypes and BW and body measurements

Association analyses were conducted between the novel SNP c.149G>A and BW and body measurements in Madura cattle (Table 3). The results showed that SNP c.149G>A had no significant effect on BW and body measurements (p > 0.05). Descriptively, the AA genotype showed the highest values for BW (312.60 ± 74.94), WH (125.20 ± 5.26), BL (128.00 ± 6.21), CG (160.80 ± 15.53), HH (124.60 ± 4.72), and HL (42.00 ± 1.73), compared to other genotypes. Meanwhile, the GG genotype had the highest mean HW value compared with the GA and AA genotypes, although the difference was not statistically significant. The lack of significant associations may be attributable to the small sample size. However, the potential SNP (c.149G>A) needs to be confirmed through further study with a larger sample size of Madura cattle.

**Table 3:** Association between novelty SNP (c.149G>A) genotypes with body weight and body measurements.

Parameters	GG (n = 11)	GA (n = 29)	AA (n = 5)	p*-*value
BW (kg)	242.50 ± 75.43	258.67 ± 58.90	312.60 ± 74.94	0.146
WH (cm)	117.09 ± 9.99	119.31 ± 7.57	125.20 ± 5.26	0.184
BL (cm)	123.00 ± 10.00	122.69 ± 11.56	128.00 ± 6.21	0.596
CG (cm)	146.45 ± 17.62	152.48 ± 11.21	160.80 ± 15.53	0.145
HH (cm)	115.55 ± 10.62	120.69 ± 6.28	124.60 ± 4.72	0.058
HL (cm)	38.64 ± 4.20	39.66 ± 3.70	42.00 ± 1.73	0.251
HW (cm)	17.27 ± 1.95	17.10 ± 1.86	17.00 ± 1.41	0.953

SNPs=Single-nucleotide polymorphisms, n = Number of samples, BW=Body weight, WH=Withers height, BL=Body length, CG=Chest girth, HH=Hip height, HL=Head length, HW=Head width

## DISCUSSION

### Expression and structure of the *LEP* gene

The *LEP* gene encodes *LEP*, a hormone expressed in various bovine tissues, including skeletal muscle, the gastrointestinal tract, pituitary gland, and mammary gland [12–14]. In cattle, this gene is located on chromosome 4 and encodes a protein consisting of 167 amino acids [13–16].

### Previously reported *LLEP* gene polymorphisms

Previous investigations have revealed multiple SNPs in exon 2 of the bovine *LEP* gene, including g.1120C>T (Pro/Ser), g.1130G>A (Arg/Gln), g.1180C>H (Arg/Cys/Ser), and g.1181G>A (Arg/His) [17]. In Ongole Grade cattle, two-point mutations – g.1047C>T (R25C) and g.1048G>A (R25H) – have also been reported in exon 2 [18]. In addition, several missense mutations in exon 3 have been documented, such as g.3011C>S, g.3257C>Y, g.3260T>Y, and g.3272T>Y [19]. The SNP g.92450765G>A has similarly been identified in Hardhenu cattle [20].

### Genotype frequencies and HWE

In the present study, the heterozygous GA genotype and G allele of the novel SNP c.149G>A were found at the highest frequencies. This is consistent with a previous study by Yadav *et al*. [20], where a single allele often dominates with a frequency exceeding 0.750. The variation in HWE status – SNP1 and SNP2 being in equilibrium while SNP3 was not – could be attributed to factors such as mutation, genetic drift, selection pressure, or population structure.

### Environmental influences and sample size limitations

Although the novel SNP did not show statistically significant associations with body measurements, environmental factors such as feeding practices and housing conditions likely influenced this outcome. The animals studied were selected for participation in a local cultural festival, where management practices varied and were not tightly standardized. Moreover, the limited number of eligible cattle contributed to a small sample size, reducing the statistical power to detect significant genotype–phenotype associations.

### Comparative findings from other cattle breeds

Despite this, the relevance of *LEP* gene polymorphisms in cattle is well established in the literature. Prior studies have reported associations between *LEP* variants and traits related to growth, carcass composition, and reproduction [12, 16, 21–23]. For instance, the g.2913C>T SNP has been associated with body measurements in weaning-age Bali cattle, with the CC genotype showing superior values compared to TT and CT [8]. Similarly, the c.239C>T (p.A80V) polymorphism has been linked to meat quality characteristics, including marbling, firmness, brightness, and grade [24]. In Chinese indigenous cattle breeds, *LEP* polymorphisms were shown to influence both BW and size indices [25].

## CONCLUSION

This study successfully identified a novel missense SNP (c.149G>A) in exon 2 of the *LEP* gene in Madura cattle, resulting in an amino acid substitution from Cys to Tyr. Three genotypes, GG, GA, and AA, were observed, with the heterozygous GA genotype showing the highest frequency. Although no statistically significant associations were found between the SNP and BW or linear body measurements, descriptive analysis indicated that the AA genotype consistently exhibited the highest average values for most growth traits, including BW (312.6 kg), wither height (WH), BL, CG, HH, and HL. The findings suggest that the c.149G>A SNP may hold potential as a genetic marker for improv-ing growth traits in Madura cattle. Its consistent association with favorable phenotypic characteristics, though not statistically significant, highlights its practi-cal relevance for future breeding strategies.

However, the study is limited by a relatively small sample size (n = 45), which may have reduced the statistical power needed to detect significant genotype–phenotype associations. In addition, environmental and management variations among the animals were not fully controlled, which could have confounded the observed outcomes.

Future research should involve larger and more diverse cattle populations to validate the effects of this SNP. Further studies should also explore gene–environment interactions and functional implications through expression analysis or protein-level assays to better understand the biological role of this mutation.

In conclusion, the discovery of this novel SNP expands current knowledge of *LEP* gene polymorphisms in Indonesian cattle breeds. While the immediate associations were not statistically conclusive, the observed trends support its potential utility in molecular-assisted selection programs, paving the way for genetic improvement in Madura cattle.

## DATA AVAILABILITY

All the generated data are included in the manuscript.

## AUTHORS’ CONTRIBUTION

KK: Conceptualization of the study, sample and data collection, and drafted the manuscript. IN and WAS: Sample and data collection. RP: Validation and drafted and edited the manuscript. RFP: Methodology and data analysis. CDN: Methodology, data analysis, and drafted the manuscript. AF: Conceptualization of the study, methodology, data analysis, validation, and drafted and revised the manuscript. All authors have read and approved the final manuscript.
